# Postoperative radiotherapy in subtotally resected recurrent WHO grade 1 meningiomas with intermediate-/high-risk molecular profiles

**DOI:** 10.1093/neuonc/noaf125

**Published:** 2025-05-29

**Authors:** Maximilian Y Deng, Sybren L N Maas, Günes Anil, Philipp Sievers, Jonathan Lischalk, Eric Zhao, Sophie Rauh, Inga Jessen, Tanja Eichkorn, Sebastian Regnery, Lukas Bauer, Thomas Held, Philipp Hoegen-Sassmannshausen, Katharina Seidensaal, Juliane Hörner-Rieber, Stefan M Pfister, Antje Wick, Wolfgang Wick, Andreas von Deimling, Klaus Herfarth, Christine Jungk, Sandro M Krieg, Jürgen Debus, Felix Sahm, Laila König

**Affiliations:** Hopp Children’s Cancer Center Heidelberg (KiTZ), Heidelberg, Germany; Department of Radiation Oncology, Heidelberg University Hospital, Heidelberg University, Heidelberg, Germany; Department of Pathology, Erasmus MC Cancer Institute, University Medical Center Rotterdam, Rotterdam, The Netherlands; Department of Pathology, Leiden University Medical Center, Leiden, The Netherlands; Department of Radiation Oncology, Heidelberg Ion-Beam Therapy Center (HIT), Heidelberg University Hospital, Heidelberg University, Heidelberg, Germany; Department of Radiation Oncology, Heidelberg University Hospital, Heidelberg University, Heidelberg, Germany; CCU Neuropathology, German Consortium for Translational Cancer Research (DKTK), German Cancer Research Center (DKFZ), Heidelberg, Germany; Department of Neuropathology, Heidelberg University Hospital, Heidelberg University, Heidelberg, Germany; Department of Radiation Oncology, Perlmutter Cancer Center at New York University Langone Health at Long Island, New York, New York, USA; Princess Margaret Cancer Centre, University Health Network, Toronto, Ontario, Canada; Department of Radiation Oncology, Heidelberg Ion-Beam Therapy Center (HIT), Heidelberg University Hospital, Heidelberg University, Heidelberg, Germany; Department of Radiation Oncology, Heidelberg University Hospital, Heidelberg University, Heidelberg, Germany; Hopp Children’s Cancer Center Heidelberg (KiTZ), Heidelberg, Germany; Department of Radiation Oncology, Heidelberg University Hospital, Heidelberg University, Heidelberg, Germany; Department of Radiation Oncology, Heidelberg Ion-Beam Therapy Center (HIT), Heidelberg University Hospital, Heidelberg University, Heidelberg, Germany; Department of Radiation Oncology, Heidelberg University Hospital, Heidelberg University, Heidelberg, Germany; Department of Radiation Oncology, Heidelberg Ion-Beam Therapy Center (HIT), Heidelberg University Hospital, Heidelberg University, Heidelberg, Germany; Department of Radiation Oncology, Heidelberg University Hospital, Heidelberg University, Heidelberg, Germany; Department of Radiation Oncology, Heidelberg Ion-Beam Therapy Center (HIT), Heidelberg University Hospital, Heidelberg University, Heidelberg, Germany; Department of Radiation Oncology, Heidelberg University Hospital, Heidelberg University, Heidelberg, Germany; Department of Radiation Oncology, Heidelberg Ion-Beam Therapy Center (HIT), Heidelberg University Hospital, Heidelberg University, Heidelberg, Germany; Department of Radiation Oncology, Heidelberg University Hospital, Heidelberg University, Heidelberg, Germany; Clinical Cooperation Unit Radiation Oncology, German Cancer Research Center (DKFZ), Heidelberg, Germany; Department of Radiation Oncology, Heidelberg Ion-Beam Therapy Center (HIT), Heidelberg University Hospital, Heidelberg University, Heidelberg, Germany; Department of Radiation Oncology, Heidelberg University Hospital, Heidelberg University, Heidelberg, Germany; Department of Radiation Oncology, Heidelberg Ion-Beam Therapy Center (HIT), Heidelberg University Hospital, Heidelberg University, Heidelberg, Germany; Department of Radiation Oncology, Heidelberg University Hospital, Heidelberg University, Heidelberg, Germany; Department of Radiation Oncology, University Hospital Düsseldorf, Düsseldorf, Germany; Clinical Cooperation Unit Radiation Oncology, German Cancer Research Center (DKFZ), Heidelberg, Germany; Department of Radiation Oncology, Heidelberg Ion-Beam Therapy Center (HIT), Heidelberg University Hospital, Heidelberg University, Heidelberg, Germany; Department of Radiation Oncology, Heidelberg University Hospital, Heidelberg University, Heidelberg, Germany; Division of Pediatric Neurooncology, German Cancer Consortium (DKTK), German Cancer Research Center (DKFZ), Heidelberg, Germany; Department of Pediatric Oncology, Hematology, Immunology and Pulmonology, University Hospital Heidelberg, Heidelberg, Germany; Hopp Children’s Cancer Center Heidelberg (KiTZ), Heidelberg, Germany; Heidelberg Institute for Radiation Oncology (HIRO) and National Center for Radiation Research in Oncology (NCRO), Heidelberg, Germany; Department of Neurology, Heidelberg University Hospital, Heidelberg, Germany; Clinical Cooperation Unit Neurooncology, German Consortium for Translational Cancer Research (DKTK), German Cancer Research Center (DKFZ), Heidelberg, Germany; National Center for Tumor Diseases (NCT), NCT Heidelberg, a partnership between DKFZ and Heidelberg University Hospital, Heidelberg, Germany; Department of Neurology, Heidelberg University Hospital, Heidelberg, Germany; Clinical Cooperation Unit Neurooncology, German Consortium for Translational Cancer Research (DKTK), German Cancer Research Center (DKFZ), Heidelberg, Germany; National Center for Tumor Diseases (NCT), NCT Heidelberg, a partnership between DKFZ and Heidelberg University Hospital, Heidelberg, Germany; Heidelberg Institute for Radiation Oncology (HIRO) and National Center for Radiation Research in Oncology (NCRO), Heidelberg, Germany; CCU Neuropathology, German Consortium for Translational Cancer Research (DKTK), German Cancer Research Center (DKFZ), Heidelberg, Germany; Department of Neuropathology, Heidelberg University Hospital, Heidelberg University, Heidelberg, Germany; Department of Radiation Oncology, Heidelberg Ion-Beam Therapy Center (HIT), Heidelberg University Hospital, Heidelberg University, Heidelberg, Germany; Department of Radiation Oncology, Heidelberg University Hospital, Heidelberg University, Heidelberg, Germany; Department of Neurosurgery, University Hospital Heidelberg, Heidelberg, Germany; Department of Neurosurgery, University Hospital Heidelberg, Heidelberg, Germany; Clinical Cooperation Unit Radiation Oncology, German Cancer Research Center (DKFZ), Heidelberg, Germany; Department of Radiation Oncology, Heidelberg Ion-Beam Therapy Center (HIT), Heidelberg University Hospital, Heidelberg University, Heidelberg, Germany; Department of Radiation Oncology, Heidelberg University Hospital, Heidelberg University, Heidelberg, Germany; CCU Neuropathology, German Consortium for Translational Cancer Research (DKTK), German Cancer Research Center (DKFZ), Heidelberg, Germany; Department of Neuropathology, Heidelberg University Hospital, Heidelberg University, Heidelberg, Germany; Hopp Children’s Cancer Center Heidelberg (KiTZ), Heidelberg, Germany; Department of Radiation Oncology, Heidelberg Ion-Beam Therapy Center (HIT), Heidelberg University Hospital, Heidelberg University, Heidelberg, Germany; Department of Radiation Oncology, Heidelberg University Hospital, Heidelberg University, Heidelberg, Germany

**Keywords:** extent of resection, integrated score, molecular diagnostics, WHO grade 1 meningioma

## Abstract

**Background:**

Meningiomas represent the most common primary intracranial tumors in adults, with World Health Organization (WHO) grade 1 typically associated with favorable outcomes following gross total resection (GTR).

**Methods:**

This retrospective study included patients with CNS WHO grade 1 meningioma and available DNA methylation profiles (*n* = 210). Clinical tumor characteristics and treatment course (eg, surgical resection, extent of resection, radiotherapy [RT]) were evaluated. Integrated Scores (InS) were calculated based on methylation family using the DKFZ brain tumor classifier, CNS WHO grading, and chromosomal losses, categorized as *low*, *intermediate*, or *high*. Survival analyses employed Kaplan–Meier and Cox regression methods, with local PFS defined as the primary endpoint.

**Results:**

In newly diagnosed cases, GTR was associated with a 93.0% 3-year progression-free survival (PFS), compared to 69.3% following subtotal resection (STR). Stratification by IntS showed that patients in the IntS-*low* group had superior outcomes: 3-year PFS of 93.4 after GTR and 77.4% after STR. In contrast, patients with IntS-*intermediate/high* profiles showed significantly worse outcomes, with PFS of 85.9% after GTR and 40.0% after STR. Following tumor recurrence, particularly those with IntS-*intermediate/high*, postoperative RT after STR may improve 3-year PFS to 88.9%, compared to much lower PFS rates in newly diagnosed cases managed without adjuvant RT after STR (3-year PFS: 40.0%).

**Conclusions:**

Our findings highlight the combined impact of both the extent of resection and molecular risk profile on prognosis in newly diagnosed cases. While conservative management is feasible in lower-risk primary cases, recurrent or higher-risk patients may benefit from early postoperative RT.

## Graphical Abstract

**Graphical Abstract:**
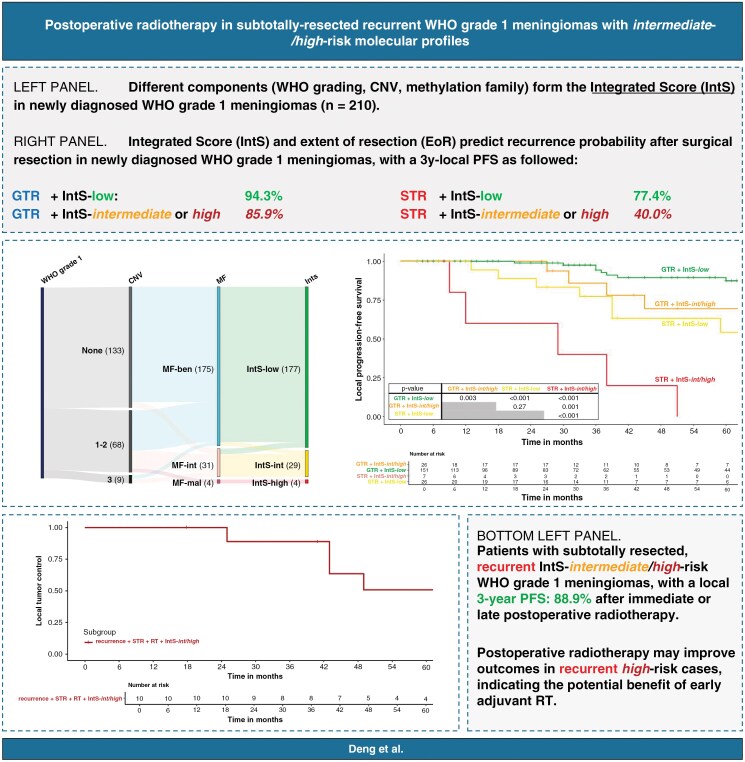

**Key Points**

1. Extent of resection and molecular profiling predict progression-free survival in newly diagnosed World Health Organization grade 1 meningiomas.
2. Postoperative radiotherapy may improve outcomes in high-risk recurrent cases after subtotal resection.

**Importance of the Study**

This study underscores the significance of combining extent of resection and molecular profiling, specifically via the Integrated Score (IntS), in predicting recurrence and progression-free survival in newly diagnosed World Health Organization (WHO) grade 1 meningiomas. While gross total resection generally results in favorable outcomes, subtotal resection is linked to higher recurrence rates, particularly in patients with IntS-*intermediate* or *high*. The study illustrates that postoperative radiotherapy (RT) may improve outcomes in recurrent or molecularly high-risk cases, indicating the potential benefit of early adjuvant RT. By integrating histological and molecular data, this study advances molecularly guided clinical decision-making, offering a more personalized approach to managing both newly diagnosed and recurrent WHO grade 1 meningiomas.

Meningiomas represent the most common primary intracranial tumor in adults, of which most are categorized as CNS World Health Organization (WHO) grade 1, associated with a favorable clinical outcome.^1^ Symptomatic patients or significantly growing meningiomas require therapeutic intervention, of which surgery is most often the preferred first choice. The risk of recurrence in patients with WHO grade 1 meningiomas after surgical resection is generally low.^2–4^ Thus, the current EANO guideline suggests that patients with incompletely resected WHO grade 1 meningiomas may not require immediate postoperative radiotherapy (RT) if no neurological symptoms persist.^2^ However, a considerable number of patients with WHO grade 1 meningiomas exhibit unexpectedly early tumor relapses, where conventional histology-based classification did not identify the increased risk of recurrence and adjuvant treatment was—thus—withheld.^1,2,5^ Furthermore, given a wide variety of institutional preferences and individualized patient circumstances, there are no standardized guidelines for the treatment of WHO grade 1 meningiomas after recurrence, in particular to the necessity of RT and its timing (eg, postoperative vs. salvage).^2,6^

In general, patients after nonradical resection were reported to show significantly worse outcomes relative to patients who underwent radical neurosurgical resection.^2,7,8^ Newly diagnosed WHO grade 1 meningiomas in the NRG Oncology/RTOG 0539 trial, referred to as *low-risk*, were directed to observation only after surgical intervention regardless of gross total (GTR) or subtotal resection (STR) was performed.^7^ Patients with WHO grade 1 meningiomas presented a 5-year progression-free survival (PFS) of 94.3% after GTR, versus 72.7% after STR.^7^ The subtotally resected WHO grade 1 patients were further compared with RTOG 0539 study patients with WHO grade 2 meningiomas following GTR and additional adjuvant RT with 54 Gy in 30 fractions: 5-year PFS was superior in the latter group (WHO grade 2, GTR + RT) with 94.1% versus 72.7 (WHO grade 1, STR). Notably, no adverse events beyond grade 2 were reported for WHO grade 2 patients who received RT after GTR.^6^ In conclusion, the NRG Oncology/RTOG 0539 study group has raised the valid question regarding optimal management in the subcohort of WHO grade 1 meningiomas following STR which could potentially benefit from adjuvant therapy.^7^ The same issue regarding the need for adjuvant treatment (eg, RT) is even more pronounced in the context of tumor recurrence after STR.

Over the past decade, molecular profiling of brain tumors has been integrated into the updates of the CNS WHO classification, offering enhanced granularity for the classification, grading, and prognostic assessment of tumor types and subtypes.^1^ For meningioma, molecular criteria differentiating low-risk from high-risk cases are limited to the presence or homozygous loss of CDKN2A/B and a TERT-promotor mutation as criteria for a WHO grade 3 tumor. However, different molecular classification systems have been established for risk prediction in meningioma, leveraging DNA methylation profiling, recurring somatic short variants, copy-number variants (CNVs), and differentially expressed genes, or a combination of molecular and histological criteria.^5,7,9–12^ Our institutionally established score, known as the Integrated Score (IntS), combines histological grading, CNVs, and DNA methylation profiles—generating a 3-tiered score which was demonstrated to improve the precision in risk stratification.^11,13^ Although the identification of molecular risk factors has been retrospective in design, independent validation in prospective cohorts is essential to advance the integration of molecularly-based risk prediction in meningiomas, which was post hoc realized for the EORTC 22042–26042 trial on WHO grades 2 and 3 meningiomas undergoing adjuvant high-dose RT or independent single-institutional studies.^13–16^ A comprehensive study by Chen et al. demonstrated the utility of a targeted 34-gene expression biomarker in predicting treatment response after postoperative RT and overall outcome in meningioma patients, with the potential to optimize postoperative treatment strategies for up to 30% of all patients.^7^ Remarkably, postoperative RT demonstrated a significant benefit in a subcohort of unfavorable WHO grade 2 meningiomas (molecular high-risk + any extent of resection, molecular intermediate-risk + STR), with a 5-year local freedom from recurrence (LFFR) of 72.5% compared to 15.6% after surgery alone. Building on these findings, a subsequent study demonstrated that the targeted gene expression biomarker enables a more refined risk stratification of clinically low-risk meningiomas, defined as WHO grade 1 tumors with gross total resection (GTR) and newly diagnosed WHO grade 2 tumors with GTR. Notably, 19.8% of these tumors exhibit high-risk molecular characteristics, indicating an increased recurrence risk.^17^

Our study presents the clinical outcome of 210 patients with newly diagnosed WHO grade 1 meningiomas with respect to the extent of resection (EoR) and IntS. Furthermore, our study aims to investigate a potential benefit of postoperative RT in higher-risk recurrent WHO grade 1 meningiomas.

## Methods

### Patient Cohort and Clinical Characteristics

The retrospective study cohort was assembled and screened for patients with CNS WHO grade 1 meningioma and available DNA methylation profiles (*n* = 210) were retrospectively identified and clinical characteristics (eg, age at diagnosis, date of diagnosis, sex), tumor characteristics (location, size, available histological and molecular features), and the course of treatment (including surgical resection, RT) were assessed. Patients who underwent adjuvant RT after initial resection were excluded. The extent of tumor resection was assessed after neurosurgical procedures via first postoperative MRIs and surgical reports, and classified as GTR and STR.^2,8,18,19^ In cases where there was a discrepancy between the postoperative MRI and the surgical report, postoperative MRI was prioritized as the most objective assessment tool. If the MRI findings were inconclusive—particularly following re-resection—postoperative treatment strategies were determined through our multidisciplinary tumor board. The study was approved by the Independent Ethics Committee of the Medical Faculty Heidelberg (S-293/2022).

### DNA Methylation Profiling and Integrated Model Score

Genome-wide DNA methylation profiles were generated using the Illumina Infinium HumanMethylation450 (450k) and MethylationEPIC (EPIC) array (Illumina) at the Genomics and Proteomics Core Facility of the DKFZ. All computational analyses were performed in R version 3.4.1 (R Development Core Team, 2018), as previously described.^5,11,20^ Meningioma methylation class families were determined by the highest scoring meningioma family score as obtained from the v12.8 DKFZ brain tumor classifier at www.molecularneuropathology.org. The meningioma methylation classes—as initially presented in Sahm et al.^5^—were used in the study. Losses over 5% of the chromosomal arm were determined as relevant.^21^ Further, Integrated Scores (InS) were calculated by summarizing the respective scores of methylation family (range: 0–4), CNS WHO grading (range: 0–2), and chromosomal losses of 1p, 6q, and/or 14q (range: 0–3).^11^ The IntS was defined as: low (0–2), intermediate (3–5), and high (>5), as previously described.^11,22^ In recurrent cases, molecular testing was performed on the recurrent tumor tissue to generate the IntS.

### Reclassification Using the UCSF Meningioma Classifier

To annotate cases with UCSF meningioma subtypes (hypermitotic, immune-enriched, or Merlin-intact),^23^ a linear support vector machine was trained using the 150-patient discovery cohort, following the approach described by Choudhury et al.^23^ and implementing code from https://zenodo.org/records/6353877. To ensure comparability between the Illumina MethylationEPIC (850k) array and the HumanMethylation450 (450k) array, the set of CpG probe sites was reduced from 2000 to 1242, retaining only those represented on both array types. This model was then applied to the 565 test cases from Choudhury et al., achieving consistent classifications in 97.4% of samples.^23^ Finally, the model was used to classify the cases in the cohort into 1 of the 3 subtypes: hypermitotic, immune-enriched, or Merlin-intact.

### Survival Analysis and Statistical Considerations

For patients after surgical monotherapy, PFS was defined from surgical resection until tumor progression.^24^ For relapsed patients who received RT, local PFS was defined from completion of RT until tumor progression. Follow-up MRIs were assessed according to current guidelines of the Response Assessment in Neuro-Oncology Working Group (RANO).^24^ Patients lost to follow-up were censored at the date of the last follow-upPFS was estimated using Kaplan–Meier analysis. Cox proportional hazard regression was performed using Python 3.12. *P*-values below the .05 threshold were considered statistically significant.

### Toxicity Analysis and Statistical Considerations

Treatment toxicity was classified according to the NCI Common Terminology Criteria for Adverse Events (CTCAE) version 5.0 8–12 weeks after RT (acute toxicity) or at the last follow-up (late toxicity) (http://ctep.cancer.gov/protocolDevelopment/electronic_applications/ctc.htm). A *P*-value <.05 was considered significant. Radiation-induced contrast enhancement (RICE) was defined as new post-treatment contrast enhancement on MRI in the surrounding brain tissue within the 80% isodose line in accordance with RANO guidelines during the follow-up period.^25^

## Results

### Clinical Patient Characteristics and Oncological Outcome in Newly Diagnosed WHO Grade 1 Meningiomas

**Figure 1.**
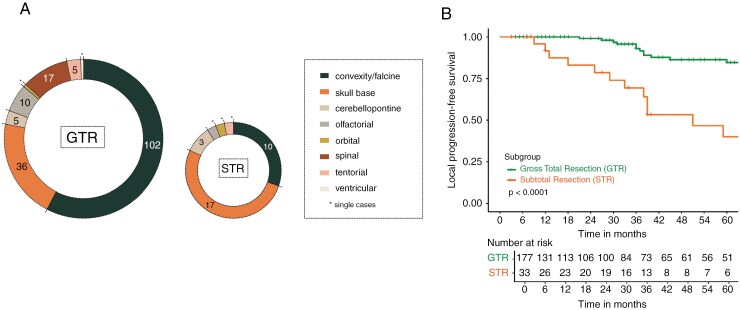
Extent of resection and local progression-free survival after gross total and subtotal resection. (A) Chord diagram illustrating the dependency of extent of resection on primary tumor location. While most convexity or falx meningioma underwent gross total resection (GTR), skull-base meningiomas were strongly represented in the subtotally resected (STR) cohort. (B) Kaplan–Meier analysis demonstrated a significantly superior local 3-year progression-free survival (PFS) with 93.0% after GTR, versus 69.3% after STR (*P* < 0.001).

In total, 210 patients with newly diagnosed CNS WHO grade 1 meningiomas and available DNA methylation data were included in our study, with a predominance of female (*n* = 138) over male patients (*n* = 72). Median age at diagnosis was 57 years (range: 8–93 years). Meningiomas were predominantly located along the convexity (27.6%, *n* = 58), parasagittal/falcine (26.2%, *n* = 55) and at the sphenoid wing/sinus cavernosus (14.3%, *n* = 30) (Table 1). GTR was achieved in 84.3% (177/210), while STR was reported in 15.7% (33/210). Notably, 89.8% (141/157) of non-skull-base meningiomas underwent GTR, compared to 67.9% (36/53) in skull-base meningiomas (sphenoid wing, parasellar, petroclival, sinus cavernous meningiomas) (Fisher’s exact test, *P* = .00038, odds ratio = 0.24) (Figure 1A). None of the patients received immediate postoperative RT after initial resection. All patients were directed to regular follow-up after initial surgical resection.

**Table 1.** Summary of the patient cohort.

Patients	Overall cohort (*n* = 210) [%]
Gender	
Female	138 [65.7]
Male	72 [34.3]
Age at initial diagnosis	
Median	57
Minimum—maximum	8-93
Tumor location	
Convexity	58 [27.6]
Parasagittal/falcine	55 [26.2]
Sphenoid wing/sinus cavernous	30 [14.3]
Petroclival	17 [8.1]
Spinal	16 [7.6]
Olfactorial	11 [5.2]
Cerebellopontine	8 [3.8]
Cerebellar	7 [3.3]
(Para)sellar	6 [2.9]
Orbital	2 [1.0]
Extent of resection	
Gross total resection	177 [84.3]
Subtotal resection/macroscopic tumor (residue)	33 [15.7]
DNA methylation class	
Benign	175 [83.3]
Intermediate	31 [14.8]
Malignant	4 [1.9]
Copy-number changes	
1q loss	63 [30.0]
6q loss	30 [14.3]
14q loss	34 [16.2]
Integrated risk scoring (IntS)	
Low	177 [84.3]
Intermediate	29 [13.8]
High	4 [1.9]

Overview of the cohort characteristics, including patient age, sex, tumor location, extent of resection, and molecular characteristics (methylation family, chromosomal losses, and the Integrated Risk Score [IntS]). Numbers in brackets indicate the corresponding percentage within the cohort.

Median follow-up time was 42 months (range: 6–486 months). Local PFS after 3 years (3-year lPFS) was estimated at 93.0% for patients after GTR (95% confidence interval [CI]: 87.7%–98.6%), versus 69.3% after STR (95% CI: 52.7–91.2, *P* < .001) (Figure 1B). While GTR was significantly associated with improved lPFS (hazard ratio [HR] = 0.195, 95% CI: 0.096–0.396, *P* < .001), neither tumor location (non-skull base vs. skull base, HR = 1.944, 95% CI: 0.918–4.114, p = 0.082) nor male gender (HR = 1.661, 95% CI: 0.799–3.451, *P* = .174) were identified as significant independent risk factors for lPFS.

**Figure 2.**
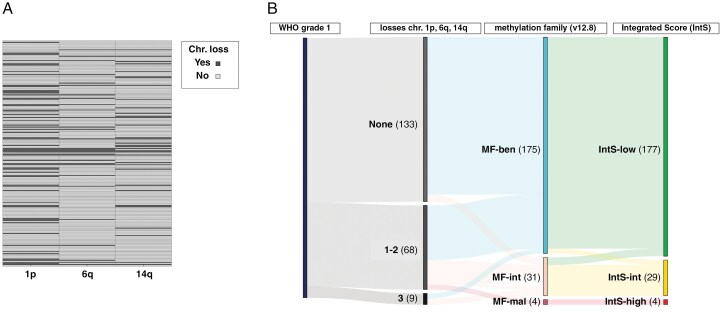
Copy-number variations and the aggregated Integrated Score (IntS). (A) Losses of chromosomal arm of 1p, 6q, and 14q were encountered in 30.0% (63/210), 14.3% (30/210), and 16.2% (34/210), respectively. Each single horizontal line represents an individual patient. (B) Sankey plot illustrating the distribution of newly diagnosed WHO grade 1 meningiomas over the different components (CNV, methylation family (MF)) of the IntS.

Methylation-based meningioma profiling was performed using the v12.8 DKFZ brain tumor classifier, which assigned 83.3% (175/210) meningiomas to the benign-, 14.8% (31/210) to the intermediate-, and 1.9% (4/210) to the malignant methylation class family. Chromosomal CNVs were frequently encountered, with loss of chromosomal arm 1p in 30.0% (63/210), loss of 6q in 14.3% (30/210), and loss of 14q in 16.2% (34/210) (Figure 2A). As previously described, the respective score for CNS WHO grade 1, CNVs, and DNA methylation family were added up, forming a 3-tiered IntS: *low* (*n* = 177/210), *intermediate* (*n* = 29/210), *high* (*n* = 4) (Figure 2B). Both IntS-*intermediate* alone (HR: 3.16, 95% CI: 1.52–6.56, *P* < .01) and a combined category of IntS-*intermediate* and IntS-*high* (HR: 3.39, 95% CI: 1.67–6.91, *P* < .01) had a significantly increased risk for local progression compared to meningiomas classified as IntS-*low*.

### Integrated Score and Extent of Resection Predict Recurrence Probability After Surgical Resection

**Figure 3.**
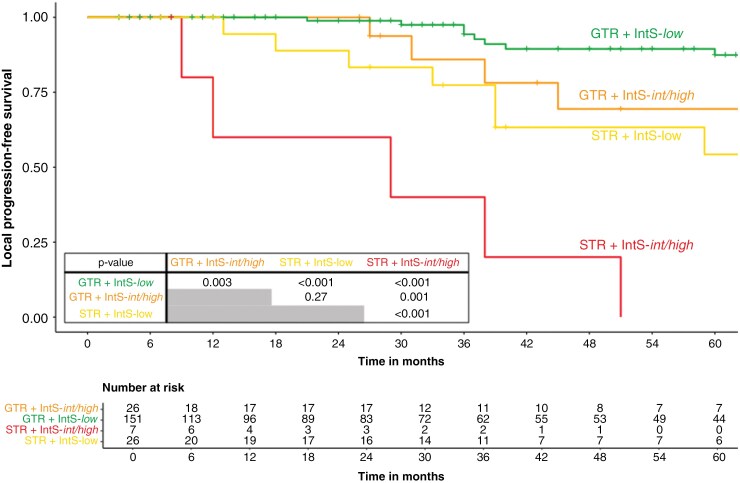
Integrated Score (IntS) and extent of resection (EoR) predict recurrence probability after surgical resection in newly diagnosed WHO grade 1 meningiomas. Patients in the IntS-*low* group demonstrated a 3-year local progression-free survival (lPFS) of 94.3% after gross total resection (GTR) and 77.4% after subtotal resection (STR). An inferior 3-year PFS was encountered in IntS-*intermediate/high*-risk patients with 85.9% after GTR and 40.0% after STR.

Risk stratification accounting for both EoR and IntS displayed substantial differences in 3-year lPFS: with 94.3% (95% CI: 89.1%–100%) in the IntS-*low* group after GTR (*n* = 151) and 77.4% (95% CI: 60.1%–99.6%) after STR (*n* = 26), versus 85.9% (95% CI: 69.5%–100%) in the IntS-*intermediate/high* group after GTR (*n* = 26) and 40.0% (95% CI: 0.14%–100%) after STR (*n* = 7) (Figure 3). No significant differences were observed in the distribution of molecular risk groups between skull-base and non-skull-base tumors (Supplementary Table 1).

### Increased Local Tumor Control Through Postoperative Radiotherapy in Recurrent Patients With Higher-Risk WHO Grade 1 Meningiomas

**Figure 4.**
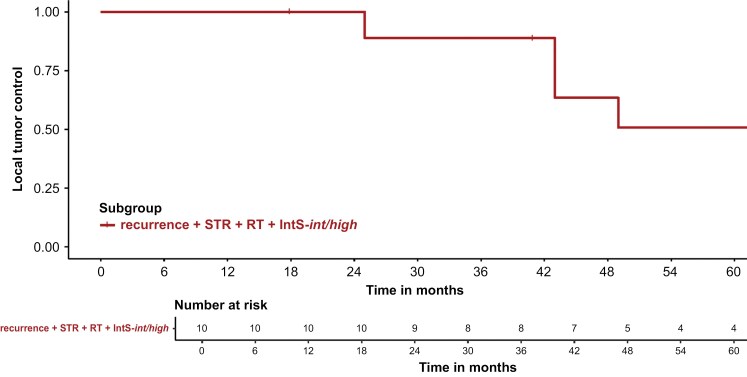
Local progression-free survival (lPFS) following postoperative radiotherapy after subtotal resection in patients with recurrent WHO grade 1 meningiomas. Patients with subtotally resected, recurrent IntS-*intermediate and high-risk* WHO grade 1 meningiomas demonstrated a local 3-year PFS of 88.9% after (immediate or late) postoperative radiotherapy.

Tumor recurrences occurred in 34/210 patients (16.2%) and were predominantly located in the skull base (sphenoid wing, sinus cavernous, petroclival, parasellar). Median time-to*-*retreatment was 39 months (range: 9–360 months) after initial surgical resection. Salvage RT was applied in 5/34 patients via fractionated external beam RT with 54 Gy in 30 fractions, using either proton (*n* = 4) or photon RT (*n* = 1). The majority of recurrences (85.3%, 29/34) were re-resected: with gross total re-resection (GTrR) in 27.6% (8/29) and subtotal re-resection (STrR) in 27.4% (21/29). The histopathological evaluation classified 27 out of 29 surgically treated recurrences as WHO grade 1, while 2 cases were designated as WHO grade 2. Histological re-evaluation of the primary tumors in these 2 patients indicated increased mitotic activity, although it did not reach the threshold required for classification as WHO grade 2 at the time. No further adjuvant treatment was applied in all patients after GTrR (8/8) and in 14.3% after STrR (3/21). Immediate postoperative RT was administered in 66.7% following STrR (14/21), while late adjuvant treatment (>3 months after re-resection) was applied in 19.0% (4/21). Median time from re-resection and commencement of RT was 2 months (range: 1–10 months). RT was mainly delivered via fractionated external beam with a median dose of 54.0 Gy (range: 50.4–59.4 Gy), comprising conventional photon RT (3D-conformal or intensity-modulated) (*n* = 6) and proton RT (*n* = 11). Both recurrences that were reclassified as WHO grade 2 were treated with a total dose of 59.4 Gy. One patient received stereotactic radiosurgery after re-resection with a single dose of 12 Gy to the surrounding 80%-isodose line (Dmax 15 Gy). lPFS following repeat surgery and—including both immediate and late—postoperative RT was estimated at 88.9% (95% CI: 70.6%–100%) after 3 years for higher-risk patients with IntS-*intermediate/high* (Figure 4).

No acute (<3 months) or late (>3 months) treatment-related toxicities exceeding CTCAE grade 2 were encountered during RT or during follow-up. Moderate fatigue and focal alopecia were the most frequent side effects during and after RT. RICE or radiation necrosis were not encountered. Detailed treatment toxicities are listed in Table 2. A comparison of the clinical characteristics between the primary, subtotal resected, and IntS-*intermediate/high* cohort and the recurrent, subtotal re-resected, and IntS-*intermediate/high* cohort is provided in Supplementary Table 2.

**Table 2.** Acute and chronic treatment toxicities during and after radiotherapy.

Toxicity	Events in % (events/overall cohort)	Mild acute events in % (CTCAE 1°–2°)	Severe acute events [%] (CTCAE 3°–4°)	Mild chronic events in %](CTCAE 1°–2°)	Severe chronic events [%] (CTCAE 3°–-4°)
Fatigue	**50.0** (9/18)	**50.0** (9/18)	-	**22.2** (4/18)	-
Headache	**27.8** (5/18)	**27.8** (5/18)	-	**16.7** (3/18)	
Dizziness	**16.7** (3/18)	**16.7** (3/18)	-	**5.6** (1/18)	-
Nausea	**20** (4/18)	**20** (4/18)	-	**-**	-
Motor deficits	**5.6** (1/18)	**5.6** (1/18)	-	**-**	-
Focal alopecia	**61.1** (11/18)	**61.1** (11/18)	-	**33.3** (6/18)	-
Sensory deficits	-	-	-	-	-
Radiation necrosis	-	-	-	-	-
Seizures	-	-	-	-	-
Hypopituitarism	-	-	-	-	-

### Reclassification Using the UCSF Meningioma Classifier

**Figure 5.**
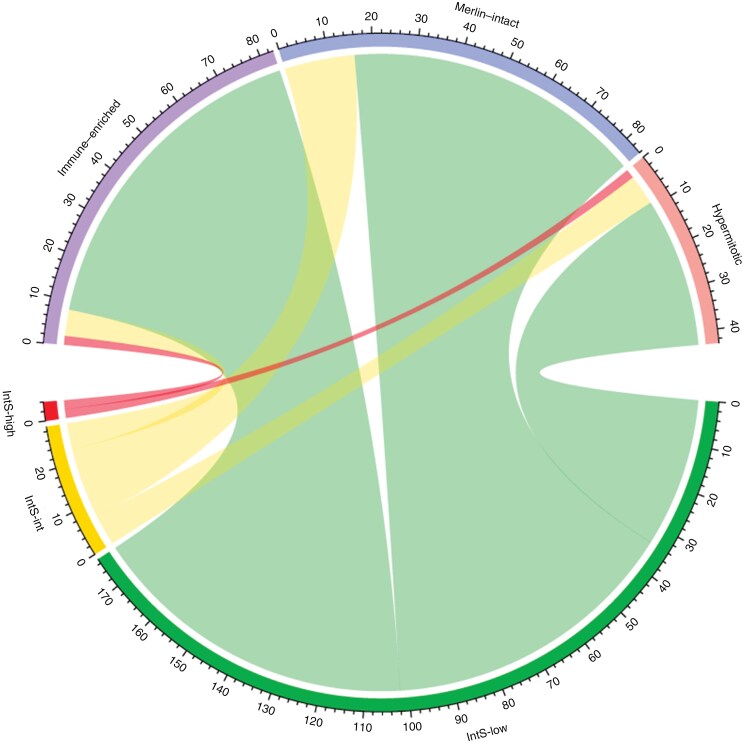
Concordance between Integrated Score (IntS) and UCSF methylation subgroup. The distribution of UCSF subgroups was analyzed in relation to the IntS. In the IntS-*low* subgroup, most cases aligned with lower-risk UCSF subgroups (42.3% immune-enriched, 38.4% Merlin-intact, 20.9% hypermitotic). *Merlin-intact* was predominant in the IntS-*intermediate* subgroup (55.2%), while *immune-enriched* and *hypermitotic* accounted for 20.7% and 24.1%, respectively. The IntS-*high* subgroup contained only 4 cases, with 2 classified as *immune-enriched* and *hypermitotic*, respectively.

**Figure 6.**
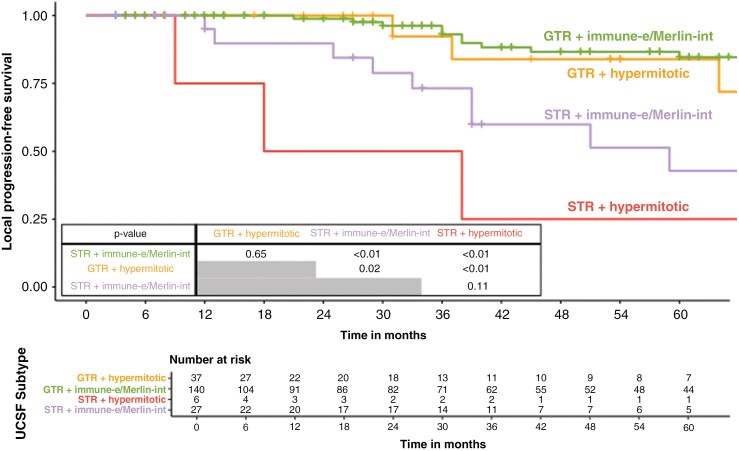
Cross-comparison using UCSF methylation subgroups for local progression-free survival after surgical resection in newly diagnosed WHO grade 1 meningiomas. Risk stratification by UCSF subgroups and extent of resection showed significant differences in 3-year local PFS: 93.1% (95% CI: 87.4%–100%) in the *Merlin-intact/immune-enriched* subgroup after gross total resection (GTR) (*n* = 140) versus 73.2% (95% CI: 55.5%–96.4%) after STR (*n* = 27), and 92.3% (95% CI: 78.9%–100%) after GTR (*n* = 37) versus 50.0% (95% CI: 18.8–100%) after STR (*n* = 6) in the *hypermitotic* subgroup.

To rule out whether our findings are prediction-model specific and if they are reproducible over different meningioma molecular risk-prediction stratifications, the cases were reclassified using the UCSF molecular grouping.^23^ Reclassification assigned 84/210 (40%) patients to the *Merlin-intact* subgroup, 83/210 (39.5%) to the *immune-enriched*, and 43/210 (20.5%) to the *hypermitotic*. The distribution of UCSF subgroups was further examined in relation to the Integrated Score (IntS-*low*, IntS-*intermediate*, IntS-*high*). The majority of cases in the IntS-*low* subgroup aligned with lower-risk UCSF subgroups, with 42.3% (75/177) classified as *immune-enriched*, 38.4% (68/177) as *Merlin-intact*, and a smaller proportion with 20.9% (34/177) as *hypermitotic*. In the IntS-*intermediate* subgroup, *Merlin-intact* was the most common classification, comprising 55.2% (16/29) of cases, while *immune-enriched* and *hypermitotic* accounted for 20.7% (6/29) and 24.1% (7/29), respectively. The IntS-*high* subgroup contained the fewest cases (*n* = 4), with 2 cases each classified as *immune-enriched* and *hypermitotic*, while none were assigned to Merlin-intact (Figure 5). Risk stratification using the UCSF classification system and EoR demonstrated substantial differences in 3-year lPFS: with 93.1% (95% CI: 87.4%–100%) in the combined *Merlin-intact/immune-enriched* group after GTR (*n* = 140) and 73.2% (95% CI: 55.5%–96.4%) after STR (*n* = 27), versus 92.3% (95% CI: 78.9%–100%) after GTR (*n* = 37) and 50.0% (95% CI: 18.8%–100%) after STR (*n* = 6) in the *hypermitotic* group (Figure 6). The distribution of UCSF subgroups in the newly diagnosed high-risk subcohort (STR + IntS-*intermediate/high*) and its corresponding recurrent counterpart (STrR + IntS-*intermediate/high*) treated with postoperative RT showed no notable differences (Supplementary Table 2). These data confirm that both molecular models can consistently predict the risk of local recurrence.

## Discussion

GTR is considered the definitive therapy for newly diagnosed WHO grade 1 meningiomas, as numerous studies have shown a strong association between EoR and rate of recurrence: a superior 5-year PFS following GTR with 94.3%, versus 72.7% after STR was demonstrated in the *low-risk* arm of the prospective RTOG 0539 trial.^2,26,27^ Resection-dependent survival outcome in our study cohort was confirmatory, as 3-year lPFS was estimated at 93.0% after GTR, and 69.3% after STR. For patients after STR or incomplete resection, current clinical guidelines typically recommend a watch-and-scan strategy, as the benefit of (immediate) postoperative RT and its timing remain subject to debate due to a lack of prospective randomized trials.^2^ However, the RTOG 0539 trial has recently reported that both newly diagnosed WHO grade 2 meningiomas after GTR and/or recurrent WHO grade 1 meningiomas (*intermediate-risk* arm) demonstrated a superior 3-year PFS with 93.8% following RT, as compared to—presumably more favorable—newly diagnosed WHO grade 1 and STR alone from the *low-risk* arm with 83.1%.^6,26^ This observation raises the question regarding an intensified adjuvant treatment in selected newly diagnosed WHO grade 1 meningiomas following STR. Currently, there is no prospective randomized trial assessing the benefit of postoperative RT in WHO grade 1 meningiomas after STR, as opposed to WHO grade 2 meningiomas following GTR where both NRG-BN003 and ROAM/EORTC-1308 phase 3 trials are in place, randomizing patients to observation versus early RT.^2,28^

A post hoc molecular analysis of the prospective EORTC 22042-26042 study has demonstrated its independent prognostic value in patients with WHO grade 2–3 meningiomas following postoperative RT with 60 Gy in 30 fractions.^13^ However, the nonrandomized design of the EORTC 22042-26042 study does not allow any conclusions on the *additional* benefit of postoperative RT in regard to survival outcome.^13^ A recent study by Chen et al. presented a targeted gene expression biomarker, which outperformed the current WHO classification in predicting tumor recurrence.^7^ Notably, unfavorable WHO grade 2 meningiomas were identified based on targeted gene expression and EOR, which were reported to benefit from postoperative RT with 5-year LFFR of 72.5%, versus 15.6% after resection alone. In particular, 20.3% of primary WHO grade 1 meningiomas were upgraded to prognostically unfavorable with a 5-year LFFR of 43.0% (high-risk + STR/GTR) and 65.1% (intermediate-risk + STR).^7^ However, immediate postoperative RT was not performed in 95.3% of those unfavorable patients with WHO grade 1 meningiomas, which is in line with our historical institutional experience (95.8%, 210/219).^7^ A follow-up study by Nguyen et al. investigated 723 clinically low-risk meningiomas (WHO grade 1 or WHO grade 2 with GTR) from patients treated with surgical monotherapy. Notably, 19.8% of clinically low-risk meningiomas exhibited high-risk molecular features, indicating an increased recurrence risk. Integrating molecular risk classification with the EOR further identified 7.1% of newly diagnosed WHO grade 1 meningiomas with GTR as having unfavorable LFFR and overall survival (OS), which may benefit from adjuvant treatment.^17^

Following the prior implementation of the Heidelberg-based IntS, the combination of both EoR and IntS as strata was demonstrated to predict 3-year lPFS in our study following surgical monotherapy with 94.3% in the most favorable constellation (IntS-*low* + GTR), versus 40.0% after STR and IntS-*int/high*.^11^ For patients with recurrent WHO grade 1 meningiomas and prognostically unfavorable IntS-*int/high* profiles, 3y-lPFS was estimated at 88.9% following subtotal re-resection (STrR) and postoperative RT (*n* = 10). This observation tentatively suggests a potential benefit of intensified adjuvant treatment, as 3y-lPFS was inferior with 40.0% in their newly diagnosed counterpart (STR + IntS-*int/high*) without RT. Although transferability of treatment decisions and survival outcomes from recurrent to primary settings are inherently limited and susceptible to biases, survival data in our recurrent cohort after postoperative RT were in accordance with the reports by RTOG 0539: 3y-PFS was estimated at 93.8% after fractionated RT with 54 Gy in 30 fractions in the *intermediate-risk* arm (recurrent WHO grade 1, newly diagnosed WHO grade 2 after GTR).^6^ No significant difference in outcome was observed following fractionated RT between both subgroups (recurrent WHO grade 1 vs. WHO grade 2 tumors + GTR).^6^ While post hoc molecular analyses of the RTOG 0539 trial are still pending, it remains unresolved if recurrent WHO grade 1 meningiomas in the *intermediate*-risk arm could be classified as higher risk (eg, IntS-*int/high*), as recurrent tumors are thought to be inherently more aggressive, as opposed to newly diagnosed WHO grade 1 meningiomas that will likely capture some tumors that would not have progressed even without RT.^7,11,27,29^ Adverse events following RT with a median dose of 54.0 Gy (range: 50.4–59.4 Gy) were limited to grades 1 and 2 in our cohort—in accordance with the reported toxicity in the *intermediate-risk arm* of RTOG 0539, suggesting that normofractionated RT with 54.0 Gy in 30 fractions is generally well tolerable.^6^

Our study should be interpreted in the context of its limitation: clinical patient data and molecular profiling were retrospectively collected and therefore inherently susceptible to biases. Treatment decisions were driven by multidisciplinary consensus and patient preferences, potentially leading to differences in patient baseline, individual risk factors, and tumor characteristics beyond those characterized, especially between newly diagnosed and recurrent meningiomas. However, given the limited feasibility of a matched-pair analysis—since WHO grade 1 meningiomas rarely receive immediate adjuvant RT—higher-risk (STR + IntS-*intermediate/-high*) recurrent WHO grade 1 meningiomas were compared with their primary counterpart, providing a closer approximation to a comparable cohort. Furthermore, detailed Simpson grading may be more advantageous to refine risk stratification—however, as it remains difficult to impossible to differentiate Simpson grading on the basis of postoperative imaging alone, a binary division into GTR/STR was preferred within the scope of our present study.^18,30^ In recurrent cases, molecular testing was performed on the recurrent tumor tissue to generate the IntS. However, potential temporal dynamics remain uncertain, as clonal evolution and molecular alterations during tumor relapse may lead to changes in molecular risk classification. Further studies are needed to elucidate the extent and potential clinical implications of these changes.

The decision of immediate adjuvant RT poses a risk of overtreating patients after STR which may not have progressed with observation alone. Au contraire, deferring adjuvant RT until tumor progression can result in additional, potentially irreversible neurological damage, and further risk of additional surgery.^2,27,29^ The implementation of molecular meningioma classification was demonstrated to refine individualized risk assessment, which may guide adjuvant RT recommendation and frequency of postoperative surveillance.^2,5,7,9–12,27,31^ While low-risk WHO grade 2 meningiomas with favorable clinical and molecular features may safely undergo postoperative surveillance rather than immediate RT, WHO grade 1 meningiomas with molecular (eg, IntS-*int/high,* UCSF methylation classifier: *hypermitotic*,^23^ 34-gene-expression: *unfavorable*^7^) and clinical higher-risk (eg, STR) features may similarly benefit from consideration of postoperative RT.^2^ Following these recent advancements in molecular meningioma classification, the Consortium to Inform Molecular and Practical Approaches to CNS Tumor Taxonomy-Not Official WHO (cIMPACT-NOW) has already suggested advanced molecular testing, and recommends assigning CNS WHO grade 2 to cases with CNS WHO grade 1 morphology when they show a combination of chromosome arm 1p deletion with 22q deletion and/or NF2 oncogenic variants—in advance of the publication of a new WHO Classification of CNS tumors.^32^

In conclusion, our study suggests that postoperative RT should be considered after subtotal resection of recurrent—and may be discussed in newly diagnosed—molecularly higher-risk WHO grade 1 meningiomas.

## Supplementary Material

noaf125_suppl_Supplementary_Tables_S1-S2

## Data Availability

All code used R 4.1.0 and publicly available packages cited in the paper. Additional data used and/or analyzed will be made available upon reasonable request.
