# Supplementary material for: Postoperative radiotherapy in subtotally resected recurrent WHO grade 1 meningiomas with intermediate-/high-risk molecular profiles
Source: Neuro Oncol. 2025 May 29;27(9):2370–81. doi: 10.1093/neuonc/noaf125 (PMC12526101; doi:10.1093/neuonc/noaf125)
Supplement: noaf125_suppl_Supplementary_Tables_S1-S2 [file noaf125_suppl_supplementary_tables_s1-s2.docx]

**Supplementary Table 1.**

|  | **IntS-low** | **IntS-intermediate** | **IntS-high** |
| --- | --- | --- | --- |
| **Non-skull base (n=157)** | 83.4% (131/157) | 14.6% (23/157) | 1.9% (3/157) |
| **Skull-based**  **(n=53)** | 86.8% (46/53) | 11.3% (6/53) | 1.9% (1/53) |

**Supplementary Table 2.**

| **Patients** | | **Primary:**  **STR alone + IntS-int/high**  **n=7** | **Recurrence:**  **STrR + RT + IntS-int/high**  **n=10** |
| --- | --- | --- | --- |
| **Gender** | | |  |
|  | female | 3 | 7 |
|  | male | 4 | 3 |
| **Age at initial diagnosis** | | | |
|  | median | 52 | 53.5 |
|  | minimum – maximum | 33-87 | 33-68 |
| **Tumor location** | | | |
|  | convexity | 2 | 4 |
|  | parasagittal/falcine | 1 | 0 |
|  | sphenoid wing/sinus cavernous | 3 | 5 |
|  | petroclival | 1 | 1 |
| **DNA methylation class** | | | |
|  | *benign* | 0 | 0 |
|  | *intermediate* | 7 | 9 |
|  | *malignant* | 0 | 1 |
| **Copy-number changes** | | | |
|  | *1q loss* | 7 | 9 |
|  | *6q loss* | 4 | 7 |
|  | *14q loss* | 5 | 8 |
| **Integrated risk scoring (IntS)** | | | |
|  | *Low* | 0 | 0 |
|  | *Intermediate* | 7 | 9 |
|  | *High* | 0 | 1 |
| **UCSF methylation subgroup (Choudhury et al.)** | | | |
|  | *Merlin-intact* | 3 | 4 |
|  | *Immune-enriched* | 2 | 2 |
|  | *Hypermitotic* | 2 | 4 |
